# The correlation between pretreatment cytokine expression patterns in peripheral blood mononuclear cells with chronic hepatitis c outcome

**DOI:** 10.1186/s12879-015-1305-1

**Published:** 2015-12-04

**Authors:** Joanna Jabłońska, Tomasz Pawłowski, Tomasz Laskus, Małgorzata Zalewska, Małgorzata Inglot, Sylwia Osowska, Karol Perlejewski, Iwona Bukowska-Ośko, Kamila Caraballo Cortes, Agnieszka Pawełczyk, Piotr Ząbek, Marek Radkowski

**Affiliations:** Department of Hepatology and Acquired Immunodeficiences, Medical University of Warsaw, Warsaw, Poland; Division of Psychotherapy and Psychosomatic Medicine, Wrocław Medical University, Wrocław, Poland; Department of Immunopathology of Infectious Diseases, Medical University of Warsaw, Warsaw, Poland; Department of Infectious Diseases, Hepatology and Acquired Immune Deficiences, Wrocław Medical University, Wrocław, Poland; Department of General Surgery and Clinical Nutrition, Medical University of Warsaw, Warsaw, Poland; Municipal Hospital of Infectious Diseases, Warsaw, Poland

**Keywords:** HCV, Antiviral treatment, PBMC, Cytokines, Gene expression

## Abstract

**Backgroud:**

Cytokine response against hepatitis C virus (HCV) is likely to determine the natural course of infection as well as the outcome of antiviral treatment. However, the role of particular cytokines remains unclear. The current study analyzed activation of cytokine response in chronic hepatitis C patients undergoing standard antiviral treatment.

**Methods:**

Twenty-two patients were treated with pegylated interferon and ribavirin. Twenty-six different cytokine transcripts were measured quantitatively in peripheral blood mononuclear cells (PBMC) before and after therapy and correlated with therapy outcome as well as with clinical and liver histological data.

**Results:**

We found that patients who achieved sustained virological response (SVR) showed higher pretreatment cytokine response when compared to subjects in whom therapy was unsuccessful. The differentially expressed factors included IL-8, IL-16, TNF-α, GM-CSF, MCP-2, TGF-β, and IP-10. Serum ALT activity and/or histological grading also positively correlated with the expression of IL-1α, IL-4, IL-6, IL-10, IL-12, IL-15, GM-CSF, M-CSF, MCP-2 and TGF-β.

**Conclusion:**

Pretreatment activation of the immune system, as reflected by cytokines transcripts upregulation, positively correlates with treatment outcome and closely reflects liver inflammatory activity.

## Background

Despite impressive progress in the therapy of chronic hepatitis C, accurate prediction of treatment outcome in an individual patient remains challenging. The well established predictive factors include viral load, genotype, quasispecies characteristics and early virological response dynamics [1–3], as well as a number of host factors like race/ethnicity, age, gender, alcohol consumption and IL-28B gene diversity [4].

The immune response against HCV is considered to be the principal determinant of the natural course of infection and it also ultimately determines the outcome of IFN-based therapies [5–7]. Various pro- and anti-inflammatory factors and virus inhibiting immune system components such as cytokines and their receptors, were subject of a number of studies. Most attention was devoted to TNF-α, IFN-γ, IL-6, IL-8, IL-10 [8–13], however, many other cytokines [14–16] as well as transcription factors [17, 18] were also analyzed. A limited number of studies addressed the issue whether pretreatment levels of cytokines are predictive of outcome. Thus, in one recent study by Par et al [8] sustained virological response (SVR) was associated with a baseline increased production of TNF-alpha and IL-6 by TLR-4 activated monocytes and decreased production of IL-4 and IL-10 by PMA activated lymphocytes. These findings are in agreements with an earlier study by Umemura et al [10] in which low pretreatment levels of IL-12 and high levels of IL-10 were found to be predictive of treatment failure. Similar results were reported by Yoneda et al [14], who after analyzing 6 different cytokines concluded that low IL-10 and high IL-12 and IL-18 levels were associated with treatment response. In another study the same group reported that high levels of eotaxin and macrophage inflammatory protein (MIP)-1beta were associated with SVR [19]. High level of serum IL-12 as a predictive factor for SVR was confirmed in yet another study [15]. Although patients differed with respect to clinical, epidemiological, and ethnical background, the resuts of the above studies suggest that markers of immune activation, in particular Th1 cytokines, correlate with positive treatment outcome. However, it was also reported that low IL-6 serum concentration may be associated with SVR, which is contrary to other studies [12].

Here we report on the activation of the cytokine response in HCV infected patients undergoing antiviral treatment with pegylated interferon (peg-IFN) and ribavirin. Twenty-six different gene transcripts were measured quantitatively in peripheral blood mononuclear cells (PBMC) before and after therapy, and correlated with clinical, virological, and histological data.

## Methods

### Patients

The study group consisted of 22 HIV-negative patients with chronic hepatitis C presenting at the outpatient clinic for antiviral treatment. Some clinical, histological and biochemical data on these patients are shown in Table 1. Eligible patients were at least 18 year-old, had detectable plasma HCV RNA level and had not been previously treated for hepatitis C. Furthermore, patients were required to have no history of decompensated liver disease, absolute neutrophil count of 1500 or more per cubic millimetre, platelet count at least 70,000 per cubic millimetre, and haemoglobin level of 12 g or more per decilitre. Apart from chronic hepatitis, patients were expected not to have any other clinical conditions which might have influenced their immune status such as acute or chronic infections, autoimmune and neoplastic diseases, nor were they receiving any immunosuppressive treatment. Patients who were current alcohol or drug abusers were excluded from the study.

**Table 1:** Some clinical, virological and biochemical characteristics of the 22 studied patients

Parameter		SVR patients (*n* = 12)	Non-responders (*n* = 10)	Statistical significance
Gender (M/F)		6/6	6/4	NS
Age (yr)^a^		46.3 ± 18.7	46.8 ± 12.8	NS
Baseline viral load (IU/ml)		1.33 × 10^6^ ± 1.28 × 10^6^	1.04 × 10^6^ ± 1.14 × 10^6^	NS
Serum ALT activity (U/L)		94.1 ± 79.9	96.4 ± 50.3	NS
Grading		2.42 ± 0.16	1.92 ± 0.17	*P* = 0.029
Staging		2.54 ± 0.23	1.88 ± 0.14	*P* = 0.053
IL28B genotype	CC	7	0	*P* = 0.005
	CT	3	7	
	TT	2	3	
Genotype	1b	7	8	NS
	3a	4	1	
	4	1	1	
Compliance^b^		8 (66 %)	7 (70 %)	
Leukocytes (10^9^/L)		5.95 ± 2.22	5.80 ± 1.95	
Neutrophils (10^9^/L)		3.28 ± 1.51	3.11 ± 1.32	

*NS* not significant

^a^All values are means ± SD

^b^ According to the “80/80/80” rule [25]

Informed consent was obtained from each patient included in the study and the study protocol followed ethical guidelines of the 2013 Declaration of Helsinki. Furthermore, the study was approved by the Ethical Committee of the Warsaw Medical University.

All patients underwent standard therapy with peg-IFN alfa-2a 180 μg once weekly (Pagasys; Hoffmann-LaRoche, Basel, Switzerland) and ribavirin (Rebetol, Schering-Plough) in the dose of 1000 mg/day or 1200 mg/day if patients body weight was <75 kg or ≥75 kg, respectively. Duration of treatment was 48 weeks in patients infected with genotypes 1 and 4, and 24 weeks in patients infected genotype 3. Following current recommendations, therapy was discontinued in patients who had HCV RNA level decrease <2 log_10_ from baseline level at 12 weeks.

The infecting HCV genotype, as determined by INNO-LiPA assay (Innogenetics, NV, Gent, Belgium), was 1b in 15 (68 %), 3a in five (23 %) and 4 in two (9 %) patients. Liver biopsy was performed within 2 months preceding therapy and liver histology was assessed using the METAVIR scoring system [20].

Viral load quantification was performed at weeks 0, 12, and 48 (Cobas Amplicor HCV Monitor Test v2.0; Roche Diagnostics). The primary end-point was a sustained virological response (SVR), defined as a negative HCV RNA level 24 weeks after the end of therapy, when tested with the above qualitative assay.

DNA samples from patients were genotyped for the IL-28B rs12979860 polymorphism with commercially available SNP genotyping platform, the TaqMan SNP genotyping assays (Applied Biosystems Inc, Foster City, CA), using the 7500 Fast real-time thermocycler. TaqMan probes and primers were identical to those published by Lagging et al. [21]. For automated allele calling SDS software (Applied Biosystems Inc.) was used.

### Detection of cytokine transcripts

For the analysis of cytokine transcripts, approximately 3x 10^5^–10^6^ PBMC were isolated from blood by centrifugation over density gradient and RNA was extracted by means of a modified guanidinium thiocyanate-phenol/chloroform technique using a commercially available kit (TRIZOL LS, Gibco/BRL).

Two microliters of reverse transcripton product (equivalent to 3 × 10^4^–10^5^ cells) were directly added into 18 μl real time PCR mix containing 0.5 μM of each primer, 1.25 μM MgCl_2_ and 2 μl Master SYBRGreen I (Fast Start DNA Master SYBRGreen I, Roche Applied Sciences) and run in Light Cycler as described before [22]. Transcripts for the following cytokines/chemokines were amplified: IL-1α, IL-1β, IL-2, IL-3, IL-4, IL-6, IL-8, IL-10, IL-12, IL-15, IL-16, IL-18, TNF-α, HLA-DR, IFNα, IFN-β, GM-CSF, M-CSF, MCP-1/CCL2, MCP-2/CCL8, MIP-1α/CCL3, MIP-1β/CCL4, RANTES/CCL5, TGF-β, IP10/CXCL10, and myxovirus resistance protein A/MxA. The primers used were described previously [22].

Histone3 rRNA, which is unaffected by cell activation state, was used to normalize expression of analyzed cytokines transcripts. The calculation of gene expression level was performed by the 2^-∆ΔC^_T_ method [23].

### Statistical analysis

All data were compared using the nonparametric Mann-Whitney *U* test and Spearman’s coefficient was used to calculate the correlations. Wilcoxon matched-pairs signed rank test was employed to compare transcript levels before and after treatment and proportions were analyzed by Fisher's exact test. *P*≤ .05 was considered to be statistically significant. Calculations were performed using GraphPad Prism version 6 for Windows (GraphPad Software, San Diego California USA). Since multiple independent tests were run simultaneously, the Benjamini–Hochberg procedure (BH step-up procedure) [24] was used to control for the false discovery rate.

## Results

Twelve patients met the criteria of early viral response (EVR) defined as ≥2 log_10_ IU/ml reduction of viral load at 12 weeks of treatment and eventually achieved SVR. The remaining 10 patients were HCV RNA positive six months after treatment and were considered non-responders.

None of the patients had to be withdrawn from therapy due to adverse events, but both Peg-IFN and ribavirin dosage had to be reduced at some time point in a large proportion of patients. However, when adherence was measured according to the “80/80/80” rule [25] over 60 % patients complied (Table 1).

The relationship of SVR with demographic, virologic, and clinical characteristics is shown in Table 1. SVR patients differed from nonresponders with respect to the presence of IL28B genotype CC (*p* = 0.005) and demonstrated higher histological activity reflected by grading score (*p* = 0.029), while the difference in histological staging was borderline (*p* = 0.053). Baseline viral load and genotype as well as patients' age and gender were not significantly different in SVR patients and non-responders.

**Fig. 1:**
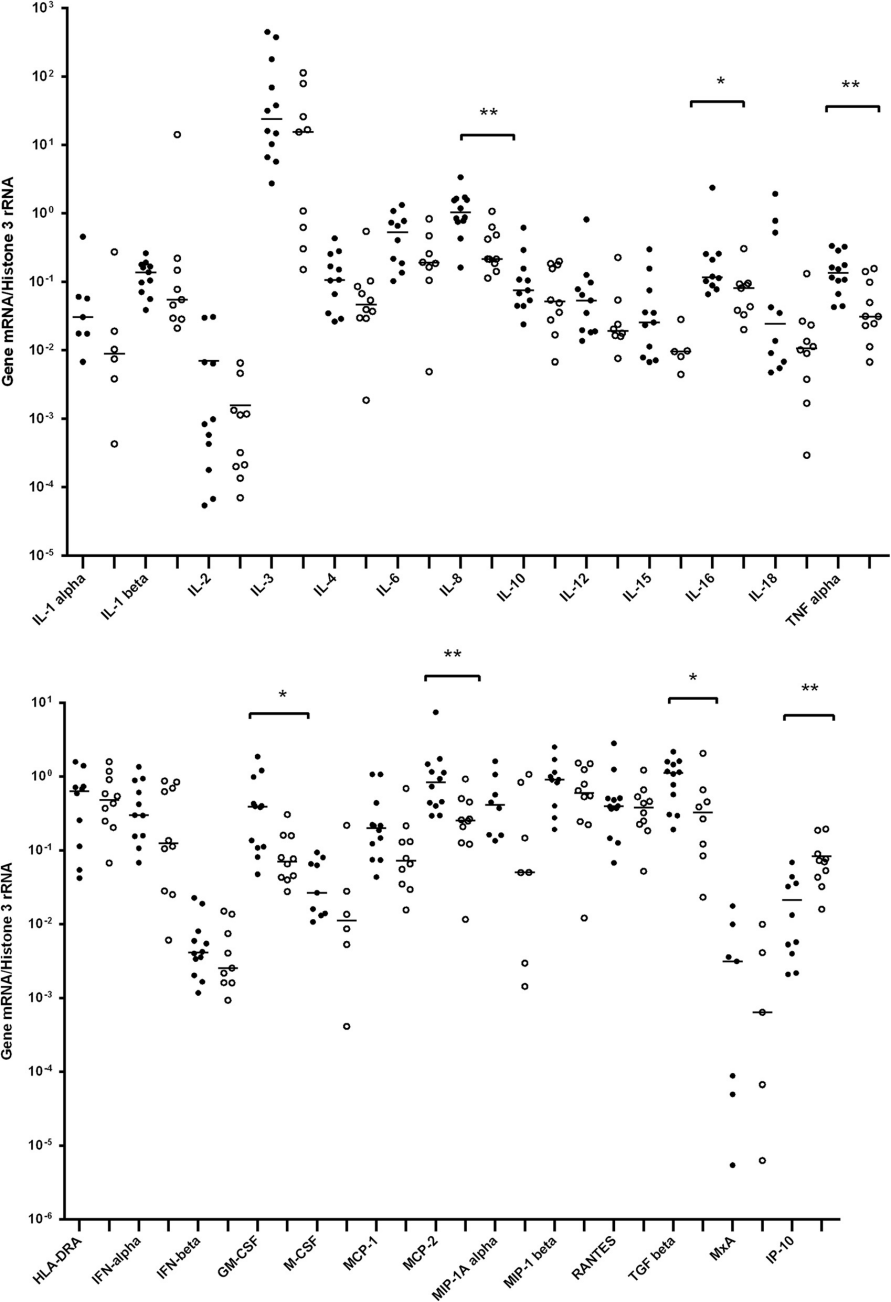
Real-time PCR analysis of gene transcripts in peripheral blood mononuclear cells (PBMC) from HCV-infected patients prior to antiviral treatment. Relative quantification of gene expression was calculated using the 2^−∆Δ*C*^_T_ method, and all data were normalized to histone 3 RNA. Solid circles represent patients achieving sustained virological response (SVR), and open circles represent patients who failed treatment. Horizontal lines indicate median values. Missing data represent situation in which the mRNA was below the detection limit. IL, Interleukin; TNF, Tumor necrosis factor; HLA-DR, human leukocyte antigen DR1; IFN, Interferon; IP-10, IFN-inducible protein-10; GM-CSF, Granulocyte-macrophage colony-stimulating factor; M-CSF, Macrophage colony-stimulating factor; MCP, Monocyte chemotactic protein; MIP, Macrophage inflammatory protein; RANTES, Regulated on activation normal T cell expressed and secreted; TGF, Transforming growth factor; MxA, Myxovirus resistance protein. *, statistically significant (*p* ≤ 0.05) by Mann-Whitney *U* test only; **. statistically significant (*p* ≤ 0.05) using Benjamini–Hochberg procedure to correct for multiple testing [24]

The results of quantitative expression of analyzed genes in PBMC samples drawn prior to the initiation of antiviral treatment among SVR patients and non-responders are presented in Fig. 1. Transcript levels of analyzed cytokine transcripts were higher in the former group than in the latter and the differences reached statistical significance (*p* ≤ 0.05) by Mann Whitney test for IL-8, IL-16, TNF-α, GM-CSF, MCP-2, TGF-β (Table 2). However, IP-10 transcripts were higher among non-responders. Since multiple independent tests were run simultaneously, we used the Benjamini–Hochberg procedure (BH step-up procedure) [24] to control for the false discovery rate (at level 0.05). Using this post hoc procedure for 26 simultaneous tests, only differences for IL-8, TNF-α, MCP-2 and IP-10 transcripts remained significant.

**Table 2:** Correlation between cytokine transcript levels in PBMC and liver biopsy grading scores and ALT activity in 22 patients with chronic hepatitis C prior to the initiation of antiviral therapy

Cytokine^a^	Liver histology Grading		ALT activity in serum	
	Spearman r	Statistical significance	Spearman r	Statistical significance
IL–1α	0.34	NS	0.62	0.025
IL–4	0.31	NS	0.66	0.002^b^
IL–6	0.47	0.05	0.46	0.050
IL–10	0.18	NS	0.59	0.005^b^
IL–12	0.27	NS	0.49	0.028
IL–15	0.19	NS	0.64	0.007^b^
GM–CSF	0.43	NS	0.49	0.030
M–CSF	0.67	0.010	0.56	0.040
MCP–2	0.28	NS	0.54	0.009^b^
TGF–β	0.39	NS	0.59	0.004^b^

^a^Only cytokines with *p*≤ 0.05 are listed

^b^statistically significant (*p*≤ 0.05) using Benjamini–Hochberg procedure to correct for multiple testing [24]

When the same analysis was repeated 6 months after the end of treatment, there were no significant differences between the two groups in any of the above parameters, as all analyzed transcripts fell in both SVR patients and non-responders (not shown). This drop in cytokine transcripts was significant (*p* ≤ 0.05) for IL-8 and TGF-β in the former and for IL-1α and MCP-2 in the latter group of patients. However, when correction for the multiple independent testing was applied, none of these differences remained significant.

While viral load correlated neither with liver histology nor ALT activity, there was a significant correlation between cytokine transcript levels in PBMC and liver biopsy grading scores and serum ALT activity prior to the initiation of antiviral therapy. Two transcripts (IL-6 and M-CSF) showed statistically significant correlation with grading scores and ten (IL-1α, IL-4, IL-6, IL-10, IL-12, IL-15, GM-CSF, M-CSF, MCP-2 and TGF-β) correlated with ALT serum activity. However, after application of Benjamini–Hochberg procedure, only five transcripts remained significant at the level of 0.05 (IL-4, IL-10, IL-15, MCP-2 and TGF-β).

## Discussion

The effect of IFN-based anti-HCV therapy is believed to be strongly determined by pro- and anti-inflammatory cytokines constituting part of the innate and adaptive immune response [26]. These are produced by a number of cells including hepatocytes, stellate cells as well as various immune cells infiltrating liver tissue [27]. However, they are also present in peripheral blood as soluble proteins and as intracellular gene transcripts in PBMC [28–30]. Activation of the immune system, as measured by serum cytokines and genes expression levels, is commonly regarded as an important predictor of therapy efficacy [8, 10, 11]. Nevertheless, previous approaches did not define clearly which factors are clinically important [27].

In the current study we demonstrated that patients who achieve SVR differ significantly from those in whom therapy failed with respect to pretreatment immune cell activation state. Thus, the majority of 26 analyzed transcripts were upregulated in SVR-patients and statistically significant differences were found for such important factors as IL-8, IL-16, TNF-α, GM-CSF, MCP-2, TGF-β, and IP-10. This list includes major pro-inflammatory mediators known to play a pivotal role in antiviral response. Not unexpectedly, SVR-patients had higher activity of liver inflammation activity reflected by grading scores.

Interestingly, the level of IP-10 transcript correlated inversely with positive treatment outcome. IP-10 plays a central role in liver inflammation, and it is expressed in the HCV-infected liver [31]. In several independent studies, elevated serum levels of IP-10 were found to predict the failure of IFN-α–based HCV treatment [32, 33]. This paradox was explained by showing that IP-10 in the plasma of many HCV patients is enzymatically processed to produce a IP-10 receptor antagonist [34].

Our findings are in agreement with studies showing that the level of immune activation, reflected by the concentration of soluble cytokines in serum [10, 14, 15, 19] or monocytes and lymphocytes culture supernatants [8], correlate with positive treatment outcome. However, these studies were limited to a small number of cytokines, whereas we analyzed 26 different cytokine transcripts. While our analysis of PBMC transcripts with respects to treatment outcome is largely supportive of the above findings, it is of note that Huang et al [35], using Affimetrix GeneChip failed to identify any significant differences in PBMC gene expression between SVR and non-SVR patients. Similarly, Younossi et al [36] found that SVR was associated with higher pretreatment gene expression levels of cytoplasmic transcription factors signal transducer and activator of transcription-6 (STAT6) and STAT5 and lower expression level of the cytokine chemokine ligand-3 (CCL3) and not with the level of cytokines expression.

In our study activation state of peripheral immune cells was correlated with two basic and commonly used markers of liver damage: histological grading and in particular with ALT activity in serum. Association between the severity of inflammation and fibrosis and elevation of TNF-alpha and TGF-beta has been previously reported by Neuman et al [37]. Similarly, IL-18 and IL-2 were correlated with ALT activity by others [38, 39]. Interestingly, ALT activity correlated positively with a number of pro-inflammatory mediators, but also with Th_2_ response markers IL-4 and IL-10. This is not unexpected as Cocciarelli et al [11], while measuring circulating levels of IL-2, IL-4, IL-10 and IFN-gamma, found that both Th1 but particularly Th2 associated cytokines IL-4 and IL-10 were dramatically elevated in chronic hepatitis C. Enhanced Th2 responses during chronic HCV infection were earlier reported by another group [40] and very high correlation between ALT activity and IL-4 level was found by Gramenzi et al [39].

It is widely accepted that liver infiltrating immune cells are largely responsible for the inflammation and development of chronic disease [41–43]. Koziel et al. [44] demonstrated that intrahepatic cytotoxic T lymphocytes (CTL) specific for HCV were able to produce IFN-γ, TNF-α, GM-CSF, IL-8 and IL-10. Similarly, in a study performed on chimpanzees acutely infected with HCV, the dynamics of viremia and course of infection correlated with intrahepatic accumulation of HCV antigen-specific CD4+ and CD8+ cells and with their response, as measured by interferon production [45]. Activation of cytokine production could be also direct as Li et al. demonstrated Toll-like receptor-3 dependent induction of RANTES, MIP-1α, MIP-1β, IP10, and IL-6 by double-stranded HCV RNA in cultured hepatoma cells [16].

Our findings of close correlation between the immune activation state of PBMC and liver inflammation reflected by ALT and HAI scores could indicate that the functional statuts of PBMC and cells infiltrating the liver are similar. A supporting evidence comes from studies performed in acutely infected chimpanzees demonstrating correlation between HCV-specific CD8 T-cell responses in the blood and molecular and functional markers of T-cell responses in the liver [46]. Similary, in HCV-infected liver transplant recipients, the cytokine profile of intrahepatic T cells did not differ from that obtained in peripheral blood [47]. Furthermore, a proportion of blood cell population may consist of cells trafficking between liver and the blood [48, 49].

Since PBMC contain divergent cells, the use of unseparated PBMC could complicate the interpretation of the results, as pointed out by Hou et al [1]. While PBMC subpopulations were not analyzed in detail in our study, major differences were unlikely (Table 1). Furthermore, any clinical application would benefit from the simplicity of unseparated PBMC analysis

Among our patients therapy resulted in reduction of the immune activation state as reflected by the diminished transcripts level of cytokines. These findings are in accordance with observation that even unsuccessful therapy may contribute to quenching of liver tissue inflammation in HCV-infected patients [50, 51].

In addition to the expression of cytokines, only IL28B genotype CC and histological activity were significant predictors of SVR, while such established factors as pretreatment viral load and viral genotype did not play a role. However, the latter could be simply due to the small number of cases in the study. Variations in the IL28B gene have been identified as key predictors of peg-IFN and ribavirin treatment response in independent genome-wide association studies, but these initial findings were confirmed in numerous studies [52–54]. The biological mechanism behind the influence of IL28B polymorphism on treatment outcome remains unclear, but it was found that the CC variant is associated with low-level expression of intrahepatic interferon-stimulated genes [55].

## Conclusion

In conclusion, we found that pretreatment activation of the immune system, as measured by PBMC cytokines gene transcription levels, correlates with treatment outcome and closely reflects liver inflammatory activity.
