# Erratum to: ‘The correlation between pretreatment cytokine expression patterns in peripheral blood mononuclear cells with chronic hepatitis c outcome’

**DOI:** 10.1186/s12879-016-1423-4

**Published:** 2016-03-02

**Authors:** Joanna Jabłońska, Tomasz Pawłowski, Tomasz Laskus, Małgorzata Zalewska, Małgorzata Inglot, Sylwia Osowska, Karol Perlejewski, Iwona Bukowska-Ośko, Kamila Caraballo Cortes, Agnieszka Pawełczyk, Piotr Ząbek, Marek Radkowski

**Affiliations:** 1 Department of Hepatology and Acquired Immunodeficiences, Medical University of Warsaw, Warsaw, Poland; 2 Division of Psychotherapy and Psychosomatic Medicine, Wrocław Medical University, Wrocław, Poland; 3 Department of Immunopathology of Infectious Diseases, Medical University of Warsaw, Warsaw, Poland; 4 Department of Infectious Diseases, Hepatology and Acquired Immune Deficiences, Wrocław Medical University, Wrocław, Poland; 5 Department of General Surgery and Clinical Nutrition, Medical University of Warsaw, Warsaw, Poland; 6 Municipal Hospital of Infectious Diseases, Warsaw, Poland

Unfortunately, the original version of this article [1] did not contain grant information. This has now been included below:

Grant Number: N402 586140
